# Fatigue may improve equally after balance and endurance training in multiple sclerosis: a randomised, crossover clinical trial

**DOI:** 10.3389/fneur.2024.1274809

**Published:** 2024-01-19

**Authors:** Laura Perucca, Stefano Scarano, Giovanna Russo, Antonio Robecchi Majnardi, Antonio Caronni

**Affiliations:** ^1^IRCCS Istituto Auxologico Italiano, Department of Neurorehabilitation Sciences, Ospedale San Luca, Milan, Italy; ^2^Department of Biomedical Sciences for Health, Università degli Studi di Milano, Milan, Italy

**Keywords:** multiple sclerosis, fatigue, balance, endurance training, aerobic training, balance training

## Abstract

**Introduction:**

Fatigue and poor balance are frequent and severe problems in multiple sclerosis (MS) that may interact. Endurance training is known to be effective on fatigue. This study aims to test if balance training is more effective against MS fatigue.

**Methods:**

A randomised crossover trial was run, recruiting 31 MS people (21 women; median age: 46 years, range: 30–64; median EDSS: 4, range: 2.5–5). Participants received balance and endurance training alternately (15 one-to-one sessions, 5 days/week) and were assessed before (T0), after (T1), and 30 days after treatment ended (T2). The Modified Fatigue Impact Scale (MFIS) with scores linearised through Rasch analysis was the primary outcome (the lower the measure, the better the condition, i.e., the lower the fatigue symptoms). The Equiscale balance scale and posturography (EquiTest) were used to assess balance. Linear mixed-effects models with ANOVA were used for significance testing.

**Results:**

Thirteen participants had no carryover effect and were included in the primary analysis. Fatigue significantly changed across the three time points (*F*_2,58_ = 16.0; *p* < 0.001), but no difference across treatments was found. Altogether, both treatments significantly improved the MFIS measure at T1 (95%CI: −1.24 logits; mean: −1.67 to −0.81 logits) and T2 (95%CI: −1.04; mean: −1.49 to −0.60) compared to T0 (95%CI: −0.51; mean: −0.95 to −0.08; *p* ≤ 0.001). Equiscale and posturography highlighted balance improvement after balance training but not after endurance training.

**Conclusion:**

Balance and endurance training could similarly reduce fatigue in MS patients in the short term. However, only balance training also improved balance in MS.

## 1 Introduction

Fatigue is one of the most common and disabling symptoms of multiple sclerosis (MS) (1), an inflammatory demyelinating disease of the central nervous system and the commonest cause of chronic disability of neurological origin in young adults (2).

The causes of fatigue in MS are still poorly understood (1). It could originate from the dysregulation of the immune system, with cytokines also considered to mediate fatigue, implicated in the pathogenesis of MS. Fatigue could be a side effect of several treatments often prescribed in MS, such as immunomodulatory therapies, anti-spasticity drugs, and benzodiazepines. Causes of fatigue have also been sought in sleep disorders (which range from central sleep apnoea to chronic insomnia) and depression, both common in this disease (3).

Various treatments have been proposed for ameliorating fatigue, including drugs (4), and non-pharmacological interventions (5, 6). Among the latter, exercise training, such as aerobic training, was associated with fatigue reduction (7).

A richer understanding of the possible causes of fatigue in MS seems essential for developing novel treatments. In this regard, an intriguing association between the severity of mobility impairment and fatigue has been pointed out. For example, fatigue is more severe in severely disabled patients (3) and abruptly worsens once ambulation is affected (8). This association between disability and fatigue remains even after ruling out important confounders such as depression (3). Poor walking stability is associated with higher fatigue levels (9), and static balance difficulties make fatigue more likely in MS (10).

Similarly to fatigue, gait and mobility impairments are common in MS (11, 12). Being associated with an increased risk of falling, gait and mobility impairments flag a balance problem (13, 14), putting MS people at risk of falls and related traumas (15). Hence, treatments and exercise training in the first place have been developed to improve balance in MS (16).

Interestingly, some clinical trials also linked balance and fatigue, showing that fatigue can be reduced after balance training (17, 18). A recent meta-analysis showed that compared with “treatment as usual,” exercise combining aerobic and strength training, balance exercises, and cognitive behavioural therapy demonstrated the most substantial effects on fatigue (19). In particular, balance training seemed to be the most effective treatment (19). Notably, this same meta-analysis pointed out that the total sample size of the balance treatment was small, so further research is recommended (19).

The relation between balance and fatigue has led us to investigate whether balance training reduces fatigue in MS. To this aim, a randomised crossover trial was run to compare the effects of balance and endurance training on fatigue and balance.

## 2 Methods

The current study reports a randomised crossover trial of approximately 5 months (enrolment from 01 January 2016 to 30 November 2019; ClinicalTrials.gov: NCT06051019).

**Figure 1:**
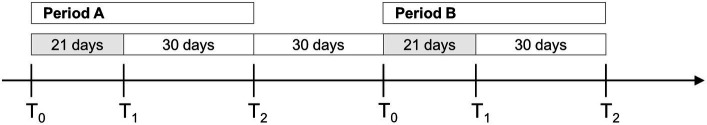
Timeline of the study. The crossover study was divided into two periods (A and B) with 30 days of washout. Participants were assessed before (T_0_), after (T_1_) and 30 days after the end of the treatment (T_2_). The grey boxes indicate training periods (21 days, 15 sessions, each lasting 90 min, 5 days/week).

Patients were randomly assigned to balance or endurance training in the first treatment period (period A) and then switched to the other type of training in the second (period B; Figure 1). In each study period, the participants completed three measurement sessions: before intervention (T_0_), at the intervention end (T_1_), and 30 days after intervention end (T_2_). The washout between the two periods was 30 days, i.e., 30 days passed between T_2_ of period A and T_0_ of period B.

Participants were recruited according to the following inclusion and exclusion criteria.

### 2.1 Inclusion criteria

- MS diagnosis according to the revised McDonald criteria (20); relapsing–remitting, and primary and secondary progressive MS forms were allowed;
- Expanded Disability Status Scale (EDSS) (21) between 2 and 6;
- Fatigue as indicated by a total score of the Modified Fatigue Impact Scale (MFIS) (22) ≥20 of 84;
- A performance on dynamic posturography [Equitest Sensory Organisation Test, SOT (14, 23, 24)] below age-matched normal values (95th percentile of control values).

### 2.2 Exclusion criteria

- Any of the following in the month before enrolment:
- An MS relapse;
- Current corticosteroids therapy because of MS;
- Change in medicines prescribed against fatigue (e.g., change in the drug type and change in dosing);
- Attending an intensive physical therapy programme (e.g., more than 45 min per session, more than once a week);
- New or active lesions on a brain or spinal cord MRI scan in the 12 months before the study enrolment;
- Angioplasty for chronic cerebrospinal venous insufficiency (25) in the 6 months before enrolment;
- Any musculoskeletal disease or any additional neurological disorder that causes by itself a balance or gait impairment;
- Any other condition causing fatigue by itself (e.g., hypothyroidism, major depression, and cardio-respiratory impairments);
- Any unstable cardiological disease (e.g., severe and uncontrolled hypertension).

As Supplementary Appendix 1 explains, the study planned to recruit at least 30 participants.

The local Ethics Committee approved the study (2016-10-25-03), and each participant gave informed consent to participate.

The CONSORT checklist for crossover trials (26) was followed.

### 2.3 Intervention and comparison treatments

Endurance (comparison) and balance (intervention) treatments consisted of 15 one-to-one physical therapy sessions, each lasting 90 min, 5 days per week.

The endurance training protocol consisted of the following eight steps:

1. Warm up with a stationary bike (no load, 10 min, 60 cycles/min).
2. Upper and lower limb stretching (10 min).
3. 10 min rest in a sitting position.
4. First exercise bout: stationary bike (15 min, 60 cycles/min); the load was modulated so that the participant’s heart rate was between 60 and 70% of the estimated maximum heart rate, and they perceived moderate fatigue on the Borg Rating of Perceived Exertion (27) (score between 11 and 14).
5. Upper and lower limb stretching (10 min).
6. 10 min rest in a sitting position.
7. Second exercise bout (same parameters as the first).
8. Upper and lower limb stretching (10 min).

The individual estimated maximum heart rate was calculated according to Tanaka et al. (28).

Balance training consisted of exercises in which keeping an upright stance was challenged. This treatment included the following five main building blocks:

1. Standing with feet together
2. Standing with closed eyes
3. Standing on unstable surfaces (e.g., foam pads and proprioceptive boards)
4. Standing while performing upper limb movements, and
5. Standing while performing head rotations.

These constituents of balance training could be combined in the same task (e.g., head-turning while standing with feet together on a foam pad) according to the participant’s ability. As an exercise for improving dynamic balance during walking, balance training included walking on a treadmill at alternating speeds (29). Finally, leg press and chest press machines were used for training trunk balance during ballistic movements of the upper and lower limbs, respectively (30). The physical therapist independently selected the exercises appropriate to the participant’s balance ability level. In addition, they administered more challenging exercises with the participant’s improvement to progress in balance training.

### 2.4 Assessments and outcome measures

The MFIS, Equiscale, Equitest SOT, and gait speed were collected in the six assessment sessions. In addition, other general variables of clinical interest were collected at the study’s recruitment (e.g., gender, age, and EDSS score).

The MFIS Italian version (22) was the study’s primary outcome, used to measure fatigue. The MFIS is a self-completed questionnaire comprising 21 items assessing how often fatigue interferes with everyday life. Representative items were item 6, “Because of my fatigue, I have had to pace myself in my physical activities,” and item 12, “Because of fatigue, I have been less motivated to do anything that requires thinking.” Respondents were asked to rate each item on five categories from 0, “never,” to 4, “almost always.” The MFIS score ranged from 0 to 84, with higher scores indicating more fatigue. For the current study, the MFIS individual scores were turned into an interval measure running a Rasch analysis (31–33) using the calibrations of items from a previous study (34).

For the primary analysis, a measure reflecting the participant’s overall fatigue was obtained from the 0 to 84 MFIS total score. However, dimensionality reduction methods highlighted a “physical” dimension and a “cognitive” dimension in the MFIS questionnaire to the point that two distinct subscales of physical and cognitive fatigue can be arranged from the MFIS items (34). As a complementary analysis, item calibrations were also used to get a measure of physical and cognitive fatigue from these MFIS physical and cognitive subdomains.

Equiscale (11) is an eight items rating scale developed to measure balance in MS. Each item is scored on three categories, with high scores indicating good balance. Two illustrative items are item 2, “standing with the eyes closed,” and item 5, “picking up an object from the floor,” assessing static and dynamic balance, respectively.

The Equitest® (Neurocom International Inc., Clackamas, OR, United States) system was used to compute the SOT, a posturography test. Details on the SOT and the Equitest can be found elsewhere (14, 24, 35). Briefly, Equitest consists of two force platforms connected to electric engines. The platforms are nested in a curved screen surrounding the subject standing on the platforms. Six different balance tasks are tested in the Equitest SOT. According to the balance task, the force platforms and the surround can stay still or rotate on the sagittal plane, driven (“sway-tuned”) by the subject’s oscillations (14).

The six tasks are more and more challenging to balance, representing actual stress tests for the mechanisms involved in standing (i.e., muscular force, proprioception, vision, and vestibular afferents) (36). For example, condition 1 tests the quiet stance (i.e., participants stand still with their eyes open, feet apart, and arms along their trunk). In condition 5, the participant stands on sway-tuned platforms with closed eyes. In condition 6, the subjects stand with their eyes open while the platforms can oscillate. In addition, the surround is also sway-tuned, challenging in a way that the systems deputed to the visual regulation of balance. From the six balance tasks, a composite score is obtained from the amplitude of the centre of mass sway during standing, ranging from 0, flagging a fall, to 100, indicating perfect stability (no sway).

Gait speed was measured from the 10 m walking test (37, 38). Participants were asked to walk 14 m in a straight corridor, and the time (in seconds) the patient took to travel the central 10 m was measured with a stopwatch. The initial and terminal walking acceleration and deceleration phases were thus discarded to have a measure of walking at a constant speed. In each session, the 10 m walking test was repeated four times in alternate directions, and the mean gait speed was recorded for the analysis.

### 2.5 Data analysis and statistics

#### 2.5.1 Assessing the carryover effect

Crossover trials rely on an essential assumption: patients are in the same state at the beginning of the second treatment as they were at the beginning of the first one (i.e., there is *no carryover effect*) (39).

Avoiding a carryover effect can be challenging, especially in a rehabilitation study on training. The following solution, a new one entirely relying on the Rasch analysis, was adopted here to assess the stability of the baselines.

As described above, the MFIS, i.e., the primary outcome of the study, was subjected to the Rasch analysis, and its items’ calibrations are available (34). These calibrations allow MFIS scores to be easily turned into interval measures, whose measurement unit is the “logit” [i.e., the measurement unit of Rasch analysis (31–33)]. It must also be remembered that any measure from the Rasch analysis comes with its standard error (SE), which quantifies the measurement precision.

Given two measures from the Rasch analysis and their SE, a Change Index can be easily calculated to assess whether they are significantly different (40):

$$\mathrm{Change}\;\mathrm{Index}=\frac{\mathrm{Measure}\;1-\mathrm{Measure}\;2}{\sqrt{\left(SE_{\mathrm{Measure}\;1}^{2}+SE_{\mathrm{Measure}\;2}^{2}\right)}}$$

If this Change Index is more extreme than 1.96 or −1.96, the two measures are significantly different “at a value of *p* < 0.05.”

The Change Index was calculated for each participant between the MFIS logit measure at A-T_0_ (i.e., T_0_ of period A) and the MFIS logit measure at B-T_0_. A Change Index <−1.96 would indicate that the MFIS measure at B-T_0_ was significantly lower (and thus the respondent’s fatigue significantly less) than at A-T_0_, thus showing a carryover effect.

Due to this analysis, it was possible to split the sample of recruited participants into a subsample with stable baselines (i.e., no evidence of carryover effect at the single subject analysis) and another subsample with significantly different baselines (i.e., patients showing a carryover effect).

#### 2.5.2 Statistical modelling

Linear mixed effects models (41) were used for the statistical analysis. Regarding the primary analysis, the MFIS logit measure was inputted in the regression models as the response variable and with the session (i.e., T_0_, T_1_, and T_2_), treatment (balance vs. endurance training), and period (A vs. B) as the predictors.

The following regression models were evaluated:

- Model 0: intercept only;
- Model 1: intercept + session;
- Model 2: intercept + session + treatment and their interaction;
- Model 3: intercept + session + treatment + period and all their interactions.

The Akaike Information Criterion (AIC) difference was used for model selection. AIC differences <2 indicate that the two models are equally good. Differences >4 suggest that the model with the smallest AIC works sensibly better than the candidate model (42).

The significance of the predictors of the model was assessed with Type III ANOVA with Satterthwaite’s method. Estimated marginal means (i.e., least-squares means) (43) were used for post-hoc testing with type I error probability (set to 0.05 as customary) corrected for multiplicity according to Holm (44). The regression assumptions of residuals normality and homogeneity of variance were checked graphically. Median and 1st to 3rd quartile were used for descriptive statistics. Differences between groups were assessed with Fisher’s exact test and Wilcoxon rank sum test in the case of count and interval or ratio data, respectively.

WINSTEPS version 5.4.3.0 (Rating Scale model) was used to obtain the participants’ MFIS measures from their scores and the items’ calibration reported in Table 1 of Mills et al. (34). R 4.2.0 was used for statistics and graphics.

**Table 1:** Participants’ clinical features.

	No carryover	Carryover	Dropped out
Females versus males, N	8 versus 5	8 versus 4	5 versus 1
Age, years	52 (41–57)	41.0 (32.5–47.0)	45 (38–54)
Time since diagnosis, years	12 (6–20)	12.0 (9.75–16.50)	13 (5–27)
EDSS, score	4.0 (3.5–4.5)	4.0 (3.8–5.0)	4.75 (4.0–6.0)
Balance training *vs* endurance training in period A, N	9 versus 4	4 versus 8	3 versus 3
MFIS, logit	−0.58 (−0.87 to −0.20)	0.00 (−0.44–0.55)	−0.11 (−0.69–1.02)
MFIS physical, logit	−0.15 (−0.80 to −0.14)	0.30 (−0.28–1.04)	0.15 (−0.42–4.48)
MFIS cognitive, logit	−0.95 (−1.68–0.33)	0.06 (−0.85–0.28)	−0.39 (−1.45–1.30)
SOT composite, %	53 (47–56)	55.5 (46.5–61.5)	45 (22–69)
Equiscale, total score	13 (12–14)	12.5 (9.75–14)	14.5 (10.0–16.0)
Gait speed, m/s	1.00 (0.82–1.11)	1.16 (0.92–1.25)	1.01 (0.64–1.30)

N, number of; period A, first study period; MFIS, Modified Fatigue Impact Scale; MFIS physical, MFIS cognitive, measures from the physical and cognitive domains of the MFIS questionnaire, respectively; SOT composite, score of the Sensory Organization Test from Equitest posturography. Unless otherwise specified, data are summarised as median (1st–3rd quartile) for the “No carryover” and “Carryover” columns. Data are summarised as median and range for the “Dropped out” column due to the reduced sample size.

## 3 Results

**Figure 2:**
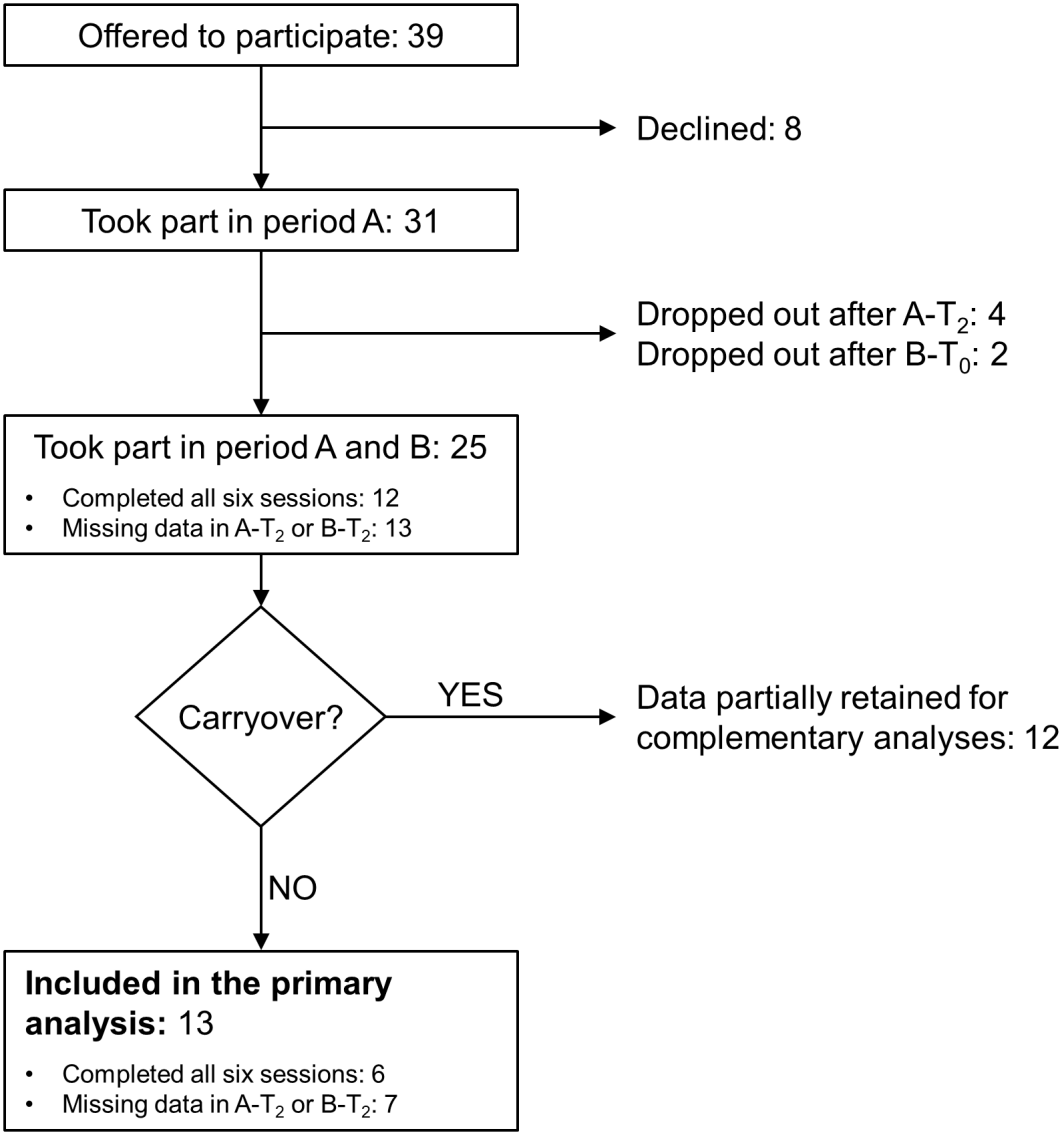
Flowchart of the study. See the text for the study's details of the study. Period A and B: first and second study period, respectively; A-T_2_: follow-up session (T_2_) of period A; B-T_0_: baseline session (T_0_) of period B; B-T_2_: follow-up session of period B.

The study screened 39 persons with MS. Eight refused to participate after the evaluation session, four dropped out after completing the third session of period A (i.e., A-T_2_), and two dropped out after completing B-T_0_ (Figure 2). Reasons for dropping out were MS relapse, hip pain at the first study period end, caregiver reasons, and change of address, causing difficulties with reaching the rehabilitation centre for attending treatments. In addition, two participants dropped out for unspecified reasons.

No apparent difference was found between dropped-out participants and the remaining 25 regarding median age, time since diagnosis, EDSS score, MFIS measure, gait speed, and gender distribution. Balance was also comparable in the two groups (Table 1).

Twelve participants completed all six evaluation sessions. For another 13, data are available for at least T_0_ and T_1_ of periods A and B.

Considering all participants with data suitable to assess the effectiveness of at least one of the two treatments, data from 31 people were available for the analysis. The clinical features of the 31 participants are reported in Table 1.

### 3.1 Assessing the baseline stability

**Figure 3:**
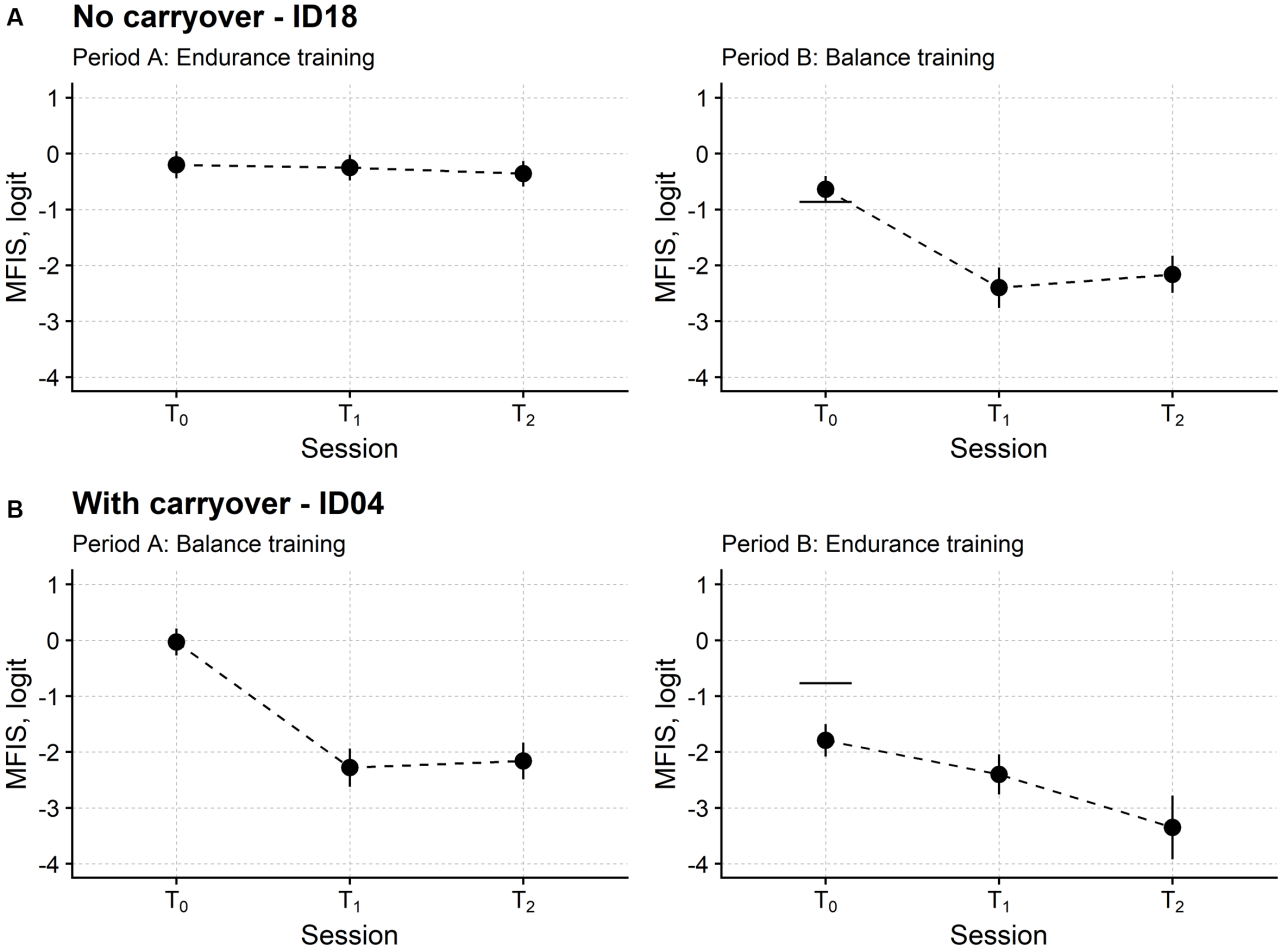
Fatigue time course in two representative participants. Subject ID18 **(A)** received endurance training in the first study period (period A) and balance training in the second (period B). Instead, Subject ID04 **(B)** received balance and endurance training. T_0_: baseline assessment session; T_1_: assessment session at treatment end; T_2_: follow-up session. Each dot marks the fatigue level measured in logit with the Modified Fatigue Impact Scale (MFIS; the lower the measure, the lower the fatigue) in a single participant. For each MFIS measure, the upward and downward vertical bars indicate one standard error (SE). The horizontal segments in the rightmost plots mark the MFIS measure from period A T_0_ minus 1.96 *SE_diff_, i.e., the SE of the difference between the MFIS measure in the two T_0_ sessions. If the MFIS measure of period B T_0_ is below this segment, fatigue is significantly lower at the beginning of period B compared with the start of period A. For ID18, the MFIS measures were not significantly different in the two baseline sessions. On the contrary, the MFIS measure of ID04 was significantly lower at T_0_ of period B compared with T_0_ of period A, indicating that fatigue had not returned to its initial baseline level at the beginning of the second study period. Therefore, the MFIS time course of ID04 showed a carryover effect.

Figure 3 reports the time course of the MFIS measure in two patients, the first and the second receiving endurance training and balance training in period A, respectively.

The patient in Figure 2 had a stable baseline: the fatigue returned at B-T_0_ (i.e., the beginning of the second phase of the study) to about the same level suffered at A-T_0_ (i.e., the study enrolment). On the contrary, the patient in Figure 2 still felt better at the beginning of the second phase, with the MFIS measure being significantly lower at B-T_0_ compared to A-T_0_.

According to this analysis, after removing dropout participants, the B-T_0_ baseline was stable in 13 patients and significantly improved in 12 patients. No participant significantly worsened their fatigue.

Different variables were compared in the two groups to understand any difference that could explain the other baseline behaviour (Table 1; see also Supplementary material for the complete analysis). Although not significant (Fisher’s exact test for count data: *p* = 0.115), treatment order was the most striking difference between the two groups. Most of the participants belonging to the stable group received balance training in the first period (8 patients out of 13). On the contrary, most patients from the group with the improved baseline started with endurance training (8 out of 12).

### 3.2 Fatigue and balance after endurance and balance training: sample analysis

#### 3.2.1 Fatigue changes after training

**Figure 4:**
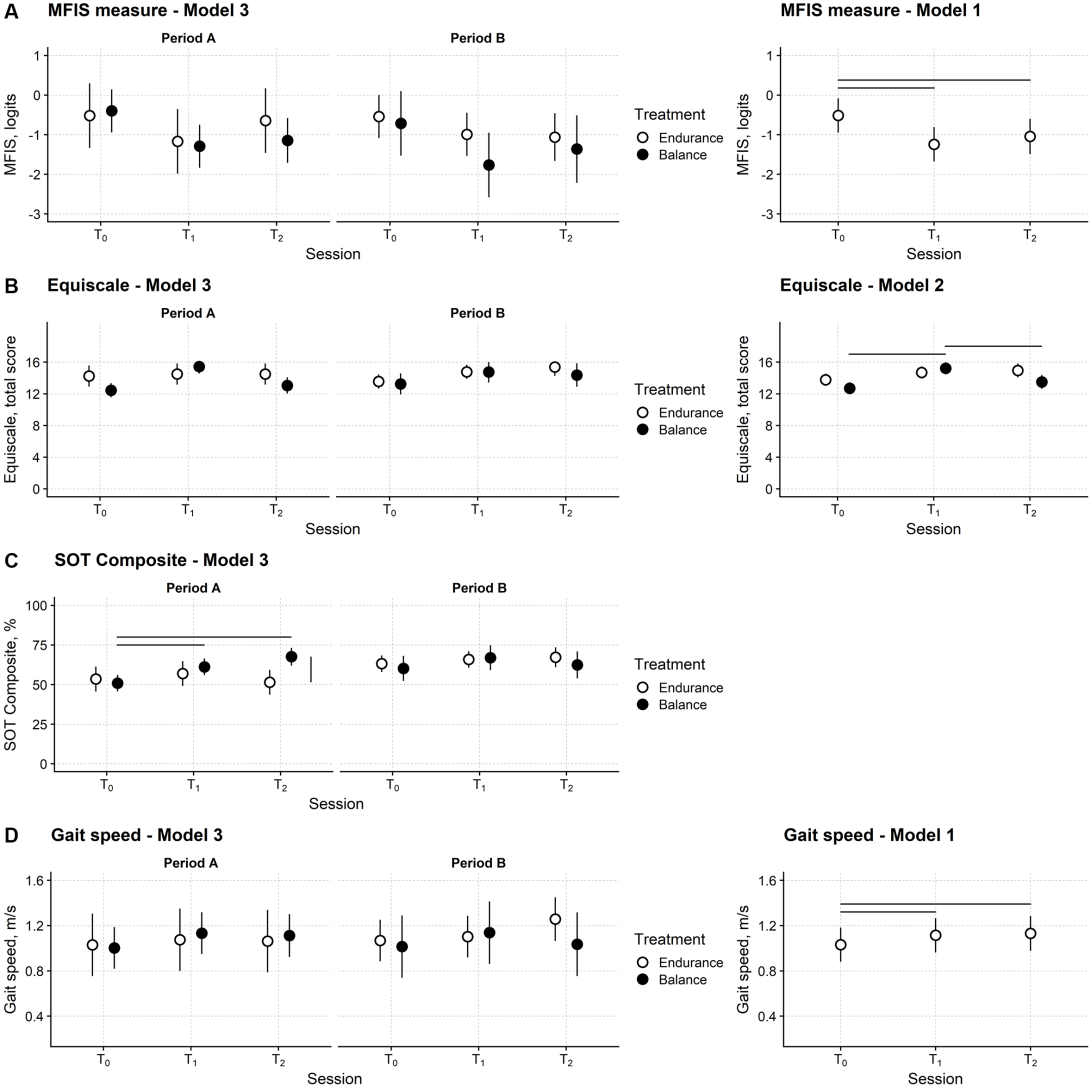
Fatigue, balance, and gait speed changes after training in the participants’ sample. Time course of the Modified Fatigue Impact Scale (MFIS) **(A)**, Equiscale **(B)**, the composite score of the Sensory Organisation Test (SOT Composite) **(C)** and gait speed **(D)**. Periods A and B: first and second study periods; T_0_: baseline assessment session; T_1_: assessment session at treatment end; T_2_: follow-up session. Black and white dots: black dots: measures from the balance study period; white dots: measures from the endurance study period. Dots report the estimated means from regression models and their 95% confidence interval (vertical bars). Horizontal bars mark significant differences between time points. The vertical bar marks a significant difference between the two treatments. The leftmost plots are from Model 3, the regression model with full predictors and fully depict the study’s results. The rightmost plots are from the models with the smallest Akaike Information Criterion (AIC), i.e., those selected for the significance analysis. Model 3 had the smallest AIC for the SOT Composite. See the Methods section for Models 1, 2, and 3 definitions.

Figure 4A shows the time course of the MFIS measure in the sample of patients showing no carryover effect.

No difference between balance and endurance training in the three sessions and two periods is apparent. The MFIS measure decreased after both types of training and remained low at the follow-up. The statistical analysis confirmed these findings.

Model 2, i.e., the model including session, treatment type, and their interaction as predictors, was the regression model with the smallest AIC (Table 2). However, the AIC difference (1.6) between model 2 and model 1, i.e., the model including only the session predictor, was negligible, and thus the simpler model 1 was preferred.

**Table 2:** Akaike information criteria of the regression models of the principal analysis.

	MFIS measure				Equiscale				SOT composite				Gait speCed			
Rank	Model	*df*	AIC	Delta	Model	*df*	AIC	Delta	Model	*df*	AIC	Delta	Model	*df*	AIC	Delta
1	Model 2	8	136.0	–	Model 2	8	247.9	–	Model 3	14	495.9	–	Model 1	5	−59.5	–
2	Model 1	5	137.6	1.6	Model 3	14	252.0	4.1	Model 2	8	515.0	19.0	Model 2	8	−58.6	0.8
3	Model 3	14	141.8	5.7	Model 1	5	254.7	6.8	Model 1	5	516.6	20.7	Model 3	14	−53.3	6.1
4	Model 0	3	159.1	23.1	Model 0	3	271.1	23.2	Model 0	3	522.3	26.4	Model 0	3	−53.1	6.4

MFIS, Modified Fatigue Impact Scale; *df*, degrees of freedom; AIC, Akaike information criterion; Delta, AIC difference; SOT composite, composite score of the sensory organization test from Equitest posturography.

ANOVA highlighted that the assessment session was a significant predictor of the MFIS measure (*F*_2,58.0_ = 16.0; *p* < 0.001). Post-hoc testing showed that compared to T_0_ (mean; 95%CI: −0.51 logits; −0.95 to −0.08 logits), the MFIS measure was significantly lower at T_1_ (−1.24; −1.67 to −0.81; *p* < 0.001) and T_2_ (−1.04; −1.49 to −0.60; *p* = 0.001). No difference was found between T1 and T_2_ (*p* = 0.175).

These findings are also valid for the physical and cognitive domains of the MFIS. In addition, complementary data analysis from the 31 patients completing at least T_0_ and T_1_ of at least one study period confirmed the above findings (Appendix 2 in Supplementary Material). Finally, in Supplementary material, an additional analysis on the subsample of participants showing a carryover effect is also reported.

#### 3.2.2 Balance changes after training

The time course of the Equiscale total score is displayed in Figure 4B.

Model 2 had the smallest AIC (Table 2), suggesting that the Equiscale score was affected only by the session and treatment of the study. ANOVA indicated that the session x treatment interaction was significant (*F*_2,57.9_ = 5.10; *p* = 0.009).

In both study periods, after balance training (but not after endurance training), Equiscale increased from T_0_ (12.7; 11.9–13.5) to T_1_ (15.2; 14.5–16.0; *p* < 0.001) and decreased to about baseline level at T_2_ (13.5; 12.6–14.4; *p* = 0.007).

For the SOT composite regression model (Figure 4C), model 3 had the smallest AIC, and the interaction between session, treatment, and period was significant (ANOVA: *F*_2,58.2_ = 4.67; *p* = 0.013).

The SOT composite score at T_1_ (61.2; 56.0–66.5) and T_2_ (67.6; 62.0–73.3) was better than at the baseline session (50.9; 45.7–56.1; *p* = 0.004 and *p* < 0.001, respectively) and similar to the Equiscale score; this improvement was found only after balance training. However, in this case, the SOT Composite score modification was found only in the first study period (Figure 4C).

Finally, for self-selected gait speed (Figure 4D), model 1 was chosen according to the AIC (ANOVA: *F*_2,57.1_ = 5.70; *p* = 0.006). Compared with T_0_ (1.03 m/s; 0.88–1.18), gait speed improved at T_1_ (1.11 m/s; 0.96–1.27; *p* = 0.015) and T_2_ (1.13 m/s; 0.98–1.28; *p* = 0.014) irrespectively of the treatment type and in the study period (Figure 4D).

#### 3.2.3 Association between balance and fatigue

As an additional analysis, linear regression was used to assess the relationship between balance measures and overall fatigue (see Supplementary Appendix 2 for full details). After ruling out the effects of session and treatment type, the SOT composite score was a significant predictor of the MFIS measure. In detail, a negative relationship was found between the SOT score and the MFIS measure, indicating that the fatigue was lower, the better the balance (slope estimate: −0.016; *p* = 0.033). On the contrary, the SOT change between sessions T_0_ and T_1_ did not predict the T_0_-T_1_ change of the MFIS measure.

## 4 Discussion

The current crossover randomised trial shows that endurance and balance training could be equally effective on fatigue in MS, physical and cognitive, in the short term. However, only balance training improved the patients’ balance.

### 4.1 How would balance training improve fatigue?

The endurance training that was effective on fatigue was an expected result based on the physiological effects of aerobic training and previous studies. Indeed, endurance training, which is used in various conditions, cardiorespiratory (45) and neurological (46), is commonly administered to improve cardiorespiratory fitness, an improvement associated with reduced fatigue (45).

Evidence of the effectiveness of endurance training against fatigue is also reported for MS (7, 47). In this regard, a Cochrane systematic review concluded that endurance training might reduce fatigue in multiple sclerosis (48).

As anticipated in the Introduction section, the conclusion that balance training could effectively improve fatigue aligns with previous works (19). For example, some clinical trials showed that the balance improvement observed after balance training could be associated with fatigue reduction (17, 49).

The benefits of balance training on fatigue can be understood considering the novel theories about the mechanisms leading to fatigue in different neurological conditions and MS.

First, it is a common experience that extra, even conscious attention is needed to avoid falls in challenging balance conditions (e.g., ice skating for the first time). This observation would suggest that a balance impairment could call for sustained attention. In support of this, the detrimental effects of a cognitive dual task on balance performance are also known in MS, e.g., Prosperini et al. (50).

However, maintaining high attention levels for a long time comes at the cost of high levels of mental fatigue (51). In turn, mental fatigue harms motor performance (52). Hence, in the vicious cycle, poor balance would be associated with attention consumption, mental fatigue, and a further deterioration of balance. Improved balance could thus reduce attentional demands, eventually mitigating cognitive fatigue.

The link between fatigue and balance can even be more subtle. For example, an association has been shown between poor gait stability, flagging poor balance while walking, and fatigue, even in MS persons with minimal neurological impairment (9). It has been proposed that when walking stability is reduced, motor performance is less predictable from stride to stride and should be inspected, checked, and corrected more often than usual. Fatigue would stem from the need for “extra computations” and refinement of the motor command for safe and effective walking. These extra computations, which could occur unconsciously, could be carried out at cortical or subcortical levels (e.g., spinal cord circuitry). With the need for a more active control of gait, the energy recovery due to a pendulum-like passive motion of the body system might be hindered (53). The idea that fatigue, cognitive and physical, would be associated with excessive neural demands (51) is in line with this hypothesis. Note that if there were not enough resources to keep up with this neural extra-work, the motor command could wane, resulting in the progressive reduction in voluntary muscular activation, i.e., central fatigue (54, 55).

A growing body of evidence suggests that chronic fatigue could be a network disorder (56), similar to chronic “nociplastic” pain (57), characterised by abnormal activity and “bad plasticity” in different brain areas (58), including sensorimotor cortices. For example, electrophysiological and imaging studies showed that fatigue in MS is associated with asymmetries between sensorimotor cortices of the two hemispheres (59) and increased activation of specific cortical areas of the motor network (60).

In line with these findings, it has been proposed that abnormal activation in the sensorimotor network can have a role in the development of fatigue in MS patients (56, 60) to the point where specific brain areas have become the target of novel treatments against fatigue. For example, repetitive transcranial magnetic stimulation, in which magnetic field pulses are administered to a brain area to induce lasting synaptic changes (61), has been applied to the primary motor cortex to improve fatigue in several diseases, including multiple sclerosis (62).

Therapeutic exercise is a powerful tool to induce plastic changes in areas of the sensorimotor network (63). Since, as reported above, this network is also involved in fatigue, a possible action mechanism of balance training to treat MS-related fatigue can be proposed.

### 4.2 It is not all about the placebo effect

The study shows that the effect sizes of balance and endurance training on fatigue are similar. The new intervention (i.e., balance training) is tested against an active treatment (i.e., endurance training), which showed effectiveness in improving fatigue in MS (47, 48).

Nevertheless, the absence of a difference when compared with an active control does not in itself demonstrates that the new treatment is effective (64, 65). Indeed, patients improving irrespectively of the administered treatment opens up the possibility that the patients get better just because of expectations or because of receiving cures.

However, two findings, both indicating that the two training modalities have produced training-specific effects in the patient sample recruited here, can be put forward against this hypothesis.

First, the current data suggest that the beneficial effects produced by endurance training could be more long-lasting than those of balance training. This, provisional, conclusion is substantiated by the fact that most participants with no carryover effect completed balance training in the first study period. On the contrary, most of the participants with carryover first received endurance training.

Second, balance, measured by a clinical scale (i.e., Equiscale) and posturography (i.e., the Equitest SOT), improved only after balance training. This last result is also important because it stresses that, in rehabilitation, specific treatments should be administered to improve specific functions. Balance training is ideal indeed for balance improvement (66).

The regression analysis between balance and fatigue measures confirms a relationship between balance impairment and fatigue (the poorer the balance, the more severe the fatigue), even after conditioning out potential confounders, such as the session and treatment type. However, no relationship was found between the amount of fatigue and balance improvement after treatments. Even if disappointing (a significant relationship would have strengthened the idea that the improvement after balance training should not be attributed to placebo only), this last result is unsurprising when considering the complexity and multi-faceted nature of the mechanisms sustaining fatigue.

In fact, as detailed in the previous paragraph, three mechanisms of action of balance training on fatigue have been proposed. A dose–response relationship between balance improvement, attention reduction, and stability improvement can be expected.

However, this relationship could be overturned by the effect induced by training on the activation of cortical brain areas. First, changes in brain activity triggered by training need not be monotonic or dose-dependent (67). In addition, brain activity changes induced by training in some brain areas and leading to better balance could worsen the imbalance between cortical nodes of the fatigue network. For example, balance training is associated with decreased corticospinal tract excitability to lower limb muscles (68, 69). However, depression along the corticospinal route is also associated with fatigue in MS (70).

### 4.3 Crossover trials for assessing therapeutic exercise effectiveness

In crossover trials, treatments are compared within the same subject, thus removing variability between subjects from the treatment effectiveness analysis (39). Since a source of unwanted variance is removed at the experimental level, compared to parallel trials, crossover trials allow for reaching a predetermined power with a smaller sample size. However, this strength comes at a cost: the assumption of no carryover must be met to make valid inferences from crossover data.

Different approaches have been proposed when a carryover affects a crossover trial. The easiest one consists in ignoring the second study period and analysing the study as if it was a parallel group trial (71). However, given the small sample size of the crossover trials, with this analysis, the trial would likely be underpowered.

The current study has applied a new method for assessing the presence and dealing with the carryover effect in crossover trials. Bypassing the carryover effect of the study has been possible because the study’s primary outcome (i.e., the MFIS questionnaire) has been validated with the Rasch analysis (31–33, 40).

More specifically, the interval measures and their standard errors obtained with the Rasch analysis from the MFIS scores have made it possible to split the whole participants’ sample into participants presenting and not presenting carryover. The subsample of MS patients with no carryover effect was eventually available for an unbiased analysis.

This novel method could be valuable for analysing crossover trials assessing non-pharmacological behavioural treatments. When interventions based on learning are investigated, a proper washout from the intervention administered in the first period can be difficult to be obtained (72).

### 4.4 Limitations and ideas for future developments

We already discussed the difficulties of demonstrating the effectiveness of a new intervention in a trial with an active treatment as the control treatment. A theoretical solution would consist of a comparison with an inactive intervention. However, defining a “true placebo treatment” in rehabilitation can be challenging.

Given the complexity of fatigue pathogenesis, the only inactive treatments for fatigue would have been stretching exercises and passive mobilisation. This solution would have been clinically (and ethically) inappropriate.

When the study was planned, it was believed that 8 weeks would have been sufficient for the washout of the first treatment before starting the second study period. However, this was not the case in about half of the recruited participants. While a limitation for crossover designs, this long-lasting beneficial effect of training in some MS patients is a clinically important finding.

To the best of our knowledge, this is the first crossover study resorting to the Rasch analysis to identify a subsample of participants without carryover. However, further research is needed to ascertain whether this methodological solution is robust enough to analyse crossover data.

## 5 Conclusion

Balance and endurance training could reduce physical and cognitive fatigue in MS patients similarly in the short term. However, while the beneficial effects of endurance training on fatigue could last longer, only balance training seems able to improve balance impairment. Balance training could thus mitigate two major problems in MS: the falling risk and fatigue.

## Data availability statement

The raw data supporting the conclusions of this article will be made available by the authors, without undue reservation.

## Ethics statement

The studies involving humans were approved by the local Ethics Committee of the Istituto Auxologico Italiano (2016-10-25-03). The studies were conducted in accordance with the local legislation and institutional requirements. The participants provided their written informed consent to participate in this study.

## Author contributions

LP: Conceptualization, Funding acquisition, Investigation, Methodology, Supervision, Writing – review & editing. SS: Data curation, Investigation, Methodology, Software, Writing – review & editing. GR: Data curation, Investigation, Writing – review & editing. AR: Conceptualization, Investigation, Supervision, Writing – review & editing. AC: Data curation, Formal analysis, Methodology, Software, Writing – original draft, Writing – review & editing.
