# Supplementary material for: Fatigue may improve equally after balance and endurance training in multiple sclerosis: a randomised, crossover clinical trial
Source: Front Neurol. 2024 Jan 19;15:1274809. doi: 10.3389/fneur.2024.1274809 (PMC10880192; doi:10.3389/fneur.2024.1274809)
Supplement: Supplementary file 1 [file Data_Sheet_1.docx]

Supplementary Material

**List of abbreviations**

- Period A and period B: first and second treatment periods;
- AIC: Akaike Information Criterion;
- EDSS: Expanded Disability Status Scale
- Equiscale: a short clinical scale returning a balance measure;
- MFIS: Modified Fatigue Impact Scale;
- SOT; Sensory Organisation Test (Equitest posturographic system);
- T_0_, T_1_ and T_2_: measurement sessions before the intervention, at the end and 30 days after.

**Appendix 1: methods details**

**Note 1.1 – Sample size estimation**

The software at page <http://hedwig.mgh.harvard.edu/sample_size/js/js_crossover_quant.html> was used for the sample size calculation.

This software gives the sample size needed for a crossover study given a type I error probability, a power value, the minimal detectable difference in means and the within-patient standard deviation (i.e. the standard deviation of repeated observations in the same individual).

The current study's type I error probability was set to 0.05 (two-sided) and power to 0.8.

Regarding the minimal change and the within-patient standard deviation, these were expressed in logit since the primary outcome was the MFIS measure obtained with the Rasch analysis from the total ordinal score.

In particular, the minimal change of interest was set to 0.5 logits since any difference smaller than 0.5 logits in the Rasch analysis framework is often considered too small to matter practically (1).

The within-patient standard deviation was also set to 0.35 logits.

One of the strengths of the Rasch analysis is that the persons' measures obtained from the questionnaires' scores come with a standard error of the estimate.

This standard error, which quantifies the precision of a measure, is the standard deviation of an imagined error distribution. In other words, if a patient were measured repeatedly under the same condition, the measured values would spread around their theoretical "true" value with a standard deviation equal to the standard error.

For the different MFIS measures, the standard error ranges from 0.23 to 1.83 logits. However, for the MFIS scores ranging from 10 to 74 (i.e. after ignoring the questionnaire extreme scores, whose measures are intrinsically less reliable (2,3)), it is < 0.35 logits.

On these premises, ten patients completing the entire study were needed.

However, it is well known that missing data are frequent with crossover trials, for example, because of the large number of assessments and the extended follow-up. Moreover, crossover trials assume no *carryover effect* (see the main text Methods section).

We assumed losing 30% of the participants and that the carryover effect could be present in another 30%. Therefore, the study eventually planned to recruit at least 30 participants.

**Appendix 2: results details**

**Note 2.1 – Baseline stability assessment: between-groups differences.**

According to the MFIS measures from the Rasch analysis (single subjects analysis), 13 participants showed no carryover effect, while the baseline significantly improved in 12. No participant significantly worsened their fatigue.

Different variables were compared in the two groups to understand any difference that could explain the other baseline behaviour (see Table 1 in the main text).

Gender distribution was comparable in the group of participants with stable (5 males and 8 females) and improved baseline (4 males and 8 females).

Age was slightly higher in stable participants (median, 1^st^ to 3^rd^ quartile: 52.0 years; 41.0 to 57.0 years) than those with improved baseline (41.0; 32.5 to 47.0), even if this difference was not statistically significant (Wilcoxon rank sum test: p = 0.077).

Even if not significant (Fisher's exact test for count data: p = 0.115), the most striking difference between the two groups was in the treatment order. Most of the participants belonging to the stable baseline group received the balance training in the first study period (8 patients out of 13). On the contrary, most patients from the group with the improved baseline started the study with endurance training (8 out of 12).

The MFIS measure at the study enrolment (A-T_0_) was also slightly lower (i.e. participants complained of less fatigue) in participants with a stable baseline (-0.58 logits; -0.87 to -0.20 logits) than in those with an improved baseline (0.00; -0.44 to 0.55). However, also in this case, the difference did not reach the significance threshold (p = 0.097).

No difference was found between the two groups for the MFIS physical and cognitive domains measures. The composite score of the SOT was also comparable in the two groups as well as the Equiscale score and gait speed. The median EDSS score was the same.

Therefore, if statistical testing is interpreted, patients with a stable baseline were older, suffered lower levels of fatigue at enrolment, and, most likely, received balance training as the first treatment when compared to patients who had maintained a significant improvement in fatigue at the beginning of the second study period.

**Note 2.2 – Fatigue after training: time course of the fatigue measures from the MFIS physical and cognitive domains**

The primary analysis of the MFIS measures showed that fatigue is significantly reduced after training irrespectively of the treatment type (balance or endurance training) and the study period. These findings are also valid for physical and cognitive fatigue assessed separately.

Table 1S reports the models' Akaike Information Criterion (AIC) with the MFIS physical domain as the response variable.

|  | **MFIS physical** | | | | **MFIS cognitive** | | | | |
| --- | --- | --- | --- | --- | --- | --- | --- | --- | --- |
| **Rank** | **Model** | **df** | **AIC** | **Delta** | **Model** | **df** | **AIC** | **Delta** |  |
| 1 | Model 1 | 5 | 174.6 | - | Model 3 | 14 | 186.7 | - |  |
| 2 | Model 2 | 8 | 176.9 | 2.3 | Model 2 | 8 | 188.2 | 1.5 |  |
| 3 | Model 3 | 14 | 183.4 | 8.7 | Model 1 | 5 | 190.3 | 3.5 |  |
| 4 | Model 0 | 3 | 190.2 | 15.6 | Model 0 | 3 | 202.9 | 16.1 |  |

Abbreviations: df: degrees of freedom; AIC: Akaike Information Criterion; Delta: AIC delta, i.e. difference to the smallest AIC.

Model 1 had the smallest AIC, and ANOVA pointed out that the session was a significant predictor of MFIS physical (F_2,58.3_ = 11.63; p < 0.001).

Posthoc tests showed that, compared to T_0_ (mean; 95%CI: -0.31 logits; -0.72 to 0.11 logits), physical fatigue significantly decreased at T_1_ (-1.20 logits; -1.61 to -0.79 logits; p < 0.001) and remained low at T_2_ (-0.82 logits; -1.26 to -0.38 logits; p = 0.031). No difference was found between physical fatigue in T_1_ and T_2_ (p = 0.063).

Regarding cognitive fatigue, model 3 had the smallest AIC. However, the AIC difference between models 3 and 2 was < 2 (Table 1S).

ANOVA of Model 2 (i.e. the model with the smallest tradeoff between parsimony and AIC) only highlighted a significant effect of "session" (F_2,58.0_ = 10.95; p < 0.001). Treatment (F_1,58.1_ = 3.18; p = 0.080) and the interaction between the session and treatment (F_2,58.1_ = 2.62; p = 0.082) were not significant.

These ANOVA findings align with the AIC difference between models 2 and 1 was two, suggesting that these two models summarise data equally well.

Also, in this case, post hoc tests highlighted that, concerning the T_0_ (-1.00 logits; -1.87 to -0.14 logits), the cognitive fatigue was significantly lower at T_1_ (-1.71 logits; -2.58 to -0.85 logits; p < 0.001) and T_2_ (-1.66 logits; -2.53 to -0.78 logits; p = 0.001). Again, no difference was found between T1 and T2 (p = 0.764).

**Note 2.3 – Fatigue after training: patients with before-after data with no baseline bias from at least one study period**

This supplementary analysis includes data free of baseline bias from 31 participants.

In detail, it comprises:

1. all the available sessions from the participants with no carryover; these data have been included in the primary analysis described in the main text,
2. sessions T_0_ and T_1_ from drop-out patients and
3. sessions T_0_ and T_1_ from period A from the subsample of participants with carryover effect.

The model with the smallest AIC was model 2 (317.2), followed by model 1 (318.9). However, the AIC difference between models 2 and 1 was 1.7; thus, model 1 is preferred.

Once again, ANOVA on model 1 confirmed that the session was a significant predictor of fatigue (F_2,93.8_ = 18.18; p < 0.001) and post hoc testing confirmed that fatigue in T_1_ (-0.93 logits; -1.31 to -0.55 logits) and T_2_ (-0.90 logits; -1.29 to -0.51 logits) was significantly lower than T_0_ (-0.21 logits; -0.59 to 0.17 logits; p < 0.001 for both comparisons).

Adding data from dropped-out participants and patients showing a carryover effect did not change the study's main conclusions.

**Note 2.4 – Association between balance and fatigue: regression analysis**

This analysis was also run on data from 31 participants. Again, only data with no baseline bias was included (see Note 2.2).

Similarly to the primary analysis reported in the main text, multiple regression models were tested, and the AIC was used for model selection.

The following regression models were assessed:

- Model 0: intercept only;
- Model 1: intercept + SOT;
- Model 2: intercept + SOT + session;
- Model 3: intercept + SOT + session + treatment;
- Model 4: intercept + SOT + session + treatment + session x treatment interaction.

SOT is the composite score of the sensory organization test, a posturographic test (see Methods section in the main text).

In all five models, the response variable was the MFIS measure.

The model with the smallest AIC was Model 3 (314.2). However, the AIC difference between Model 3 and Model 2 (316.6) was < 4, indicating that Model 3 is not "definitely better" than Model 2. For this reason and because it is simpler, Model 2 was preferred.

Significance testing on Model 2 confirmed that the session was a significant predictor of the MFIS measures. In addition, the SOT composite score was also an MFIS predictor (slope estimate: -0.016; standard error: 0.007; df: 125.0; t value: -2.16; p = 0.033). According to the slope estimate, a negative relationship linked the SOT composite score and the MFIS measure: the higher the SOT (and the better the balance), the lower the MFIS measure (and the worse the fatigue).

**Note 2.5 – Association between balance and fatigue improvement: regression analysis**

In this supplementary analysis, the T_0_-T_1_ difference of the MFIS measure was inputted as the response variable in the regression models. The T_1_-T_0_ difference of the SOT score (ΔSOT) was one of the regressors.

Note that the T_0_-T_1_ difference was calculated for the MFIS, while the T_1_-T_0_ difference was calculated for the SOT. In this way, positive MFIS and SOT deltas both flag patient improvement and results interpretation is eased.

As before, different models were contrasted with the AIC:

- Model 0: intercept only;
- Model 1: intercept + ΔSOT;
- Model 2: intercept + ΔSOT + treatment.

Data without baseline bias was included from 31 participants.

Model 2 had the smallest AIC (118.9), which was not substantially better than Model 0 (121.1).

**Note 2.6 – Complementary analysis on the subsample of participants showing a carryover effect.**

As a complementary analysis, statistical testing was also run on the sub-sample of 12 participants, showing a carryover effect at the beginning of the second phase of the trial.

Table 2S reports the models' Akaike Information Criterion (AIC) with the MFIS measures from the questionnaire total score as the response variable.

|  | **MFIS** | | | |
| --- | --- | --- | --- | --- |
| **Rank** | **Model** | **df** | **AIC** | **Delta** |
| 1 | Model 3 | 14 | 200.9 | - |
| 2 | Model 1 | 5 | 212.1 | 11.1 |
| 3 | Model 2 | 8 | 214.4 | 13.5 |
| 4 | Model 0 | 3 | 217.1 | 16.2 |

Model 3, i.e. the most complex one, comprising full predictors and interactions, is the model with the smallest AIC.

The results of ANOVA calculated on model 3 are reported below:

|  | **Sum Sq** | **Mean Sq** | **NumDF** | **DenDF** | **F** | **P-value** |  |
| --- | --- | --- | --- | --- | --- | --- | --- |
| Treatment | 0.39 | 0.39 | 1 | 52 | 0.67 | 0.417 |  |
| Session | 10.21 | 5.10 | 2 | 52 | 8.67 | 0.001 | *** |
| Period | 4.72 | 4.72 | 1 | 52 | 8.02 | 0.007 | ** |
| Treatment:Session | 1.10 | 0.55 | 2 | 52 | 0.94 | 0.398 |  |
| Treatment:Period | 4.24 | 4.24 | 1 | 12 | 7.20 | 0.020 | * |
| Session:Period | 4.46 | 2.23 | 2 | 52 | 3.79 | 0.029 | * |
| Treatment:Session:Period | 3.27 | 1.63 | 2 | 52 | 2.77 | 0.072 |  |

Abbreviations: Sum Sq: sum of squares; Mean Sq: mean square; NumDF: numerator degrees of freedom; DenDF: denominator degrees of freedom; F: F value; ***: p ≤ 0.001; **: p ≤ 0.01; *: p ≤ 0.05. Treatment: balance vs endurance training; Session: T_0_, T_1_ vs T_2_. Period: A vs B, first vs. second study period.

The session was a significant predictor of fatigue, and post hoc testing confirmed that fatigue was significantly lower at T_1_ (mean = -1.57 logits; 95% CI: -2.24 to -0.89 logits) and T_2_ (-1.64; -2.37 to -0.91) compared to T_0_ (-0.71; -1.38 to -0.03; p = 0.002 and 0.003, respectively). No difference in fatigue was found between the T_1_ and T_2_ sessions (p = 0.801).

The study's period was also a significant predictor, with fatigue in Period B (-1.60 logits; -2.28 to -0.93 logits) significantly lower than in Period A (-1.00; -1.66 to -0.34).

However, these findings should be read considering that the session times period interaction was also a significant predictor of fatigue.

First, post-hoc testing highlighted that the difference in fatigue between the three study sessions was limited to Period A.

In the first study period, fatigue was significantly lower in T_1_ (-1.28; -2.02 to -0.54) than in T_0_ (-0.03; -0.77 to 0.72; p = 0.003). Similarly, fatigue was also lower in T_2_ (-1.69; -2.48 to -0.91) compared to the first assessment (p < 0.001). No difference was found in these three comparisons in Period B (p > 0.824).

Second, differences between the two study's periods seem limited to the first session. Fatigue in session T_0_ of Period B (-1.38; -2.13 to -0.64) was significantly lower than in T_0_ of Period A (0.03; -0.77 to 0.72), pointing out the carryover effect.

The interaction between treatment and session was not significant, nor was the interaction between the three predictors, suggesting that the time course of the fatigue improvement is comparable after balance and endurance training.

On the contrary, the interaction between treatment and period was significant. Post hoc testing pointed out that, in Period A and regardless of the session, fatigue was significantly lower in the participants who received balance training (-1.88 logits; -2.96 to -0.80 logits) compared to those who received endurance training (-0.12; -0.88 to 0.64; p = 0.039).

Post-hoc also showed that, when endurance training was administered, fatigue was significantly lower in Period B (-2.31; -3.41 to -1.21) compared to Period A (-0.12; -0.88 to 0.65; p = 0.013). Again, this difference was independent of the session.

Results on the subsample of participants showing a carryover effect are complex and interpretative challenging.

In line with the primary results presented in the main text, fatigue improves after endurance and balance training and apparently to the same extent, as indicated by the statistical significance of the treatment predictor associated with no significance of the treatment times group interaction. However, these improvements are limited to the first half of the study.

This last finding can be interpreted under the carryover effect characterising these patients, confirmed in this supplementary analysis. Because of this carryover, fatigue is substantially reduced at the beginning of Period B, so there could be little room for further improvement.

The fact that fatigue is lower in Period B when endurance training is administered than in Period A lets us speculate that the balance training effects on fatigue, at least in this sub-sample of patients, last longer than those of the endurance.

The fact that fatigue was lower in Period A in those receiving balance training compared to the participants receiving endurance training could be because balance training is more effective than endurance training. Still, this difference is not strong enough to become selectively significant at T_1_ and T_2_. However, it should also be noted that an accident in the participants' randomisation cannot be ruled out.

As a final note, the provisional nature of these results must be stressed, given the reduced sample size of this supplementary analysis prompted by a discussion with Reviewer 3.

In addition, it should also be noted that Period A of the patients showing a carryover effect have already been included in the analysis reported in Note 2.3 of these Supplementary Materials.

**References**

1. Tesio L, Caronni A, Simone A, Kumbhare D, Scarano S. Interpreting results from Rasch analysis 2. Advanced model applications and the data-model fit assessment. *Disabil Rehabil* (2023)1–14. doi: 10.1080/09638288.2023.2169772

2. Tesio L, Scarano S, Hassan S, Kumbhare D, Caronni A. Why Questionnaire Scores Are Not Measures: A Question-Raising Article. *American Journal of Physical Medicine & Rehabilitation* (2023) 102: https://journals.lww.com/ajpmr/Fulltext/2023/01000/Why_Questionnaire_Scores_Are_Not_Measures__A.10.aspx

3. Tesio L, Caronni A, Kumbhare D, Scarano S. Interpreting results from Rasch analysis 1. The “most likely” measures coming from the model. *Disability and Rehabilitation* (2023) 0:1–13. doi: 10.1080/09638288.2023.2169771

**
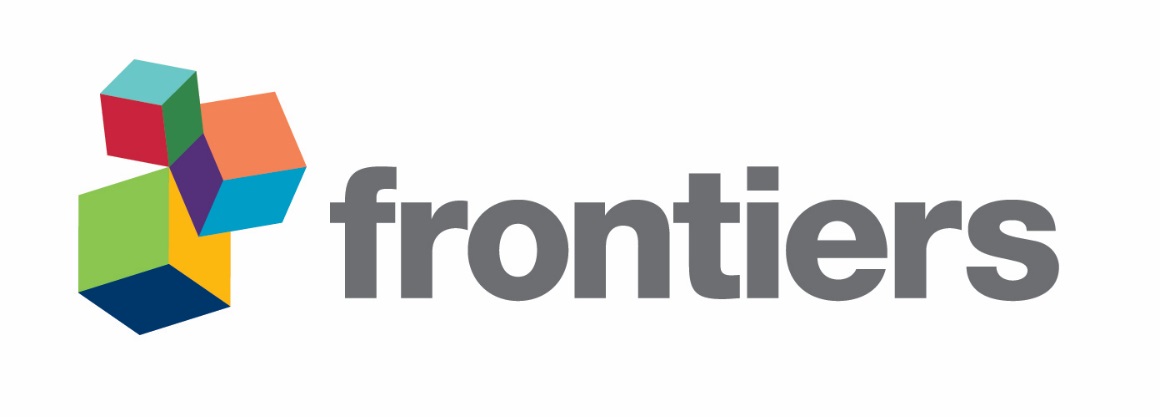
**
